# What energy infrastructure will be needed by 2050 in the EU to support 1.5°C scenarios?

**DOI:** 10.12688/f1000research.109399.1

**Published:** 2022-04-01

**Authors:** Igor Arduin, Christopher Andrey, Tobias Bossmann

**Affiliations:** 1 Artelys, Paris, 75009, France; 2 Artelys, Brussels, 1040, Belgium

**Keywords:** infrastructure, networks, 2050, decarbonisation, methane, repurposing, EU, multi-energy, renewables, power-to-gas

## Abstract

**Background: **The European Commission has settled ambitious objectives in order to reach climate neutrality by 2050. This will imply the shift from fossil fuel to low carbon energy supply and an adaptation of the energy system according to it. Electrification and production of green hydrogen are seen as structural pillars. The objective of the study is to quantify energy infrastructure needs required in various climate neutral scenarios at the 2050 time horizon.

**Methods:** The work was based on Artelys Crystal Super Grid, a tool developed at Artelys for modelling and simulating energy markets on a continental or national scale. In this study, we apply a multi-energy (i.e., power, hydrogen, methane) capacity expansion and dispatch optimisation methodology, featuring hourly and national granularity, covering the European Union plus major neighbouring countries. Several investments options are considered: storage assets, electrolysers, cross-border electricity, hydrogen and CH4 interconnections (including repurposing of CH4 infrastructures), and gas-to-power capacities.

**Results:** Important needs for cross-border electricity infrastructure appear in all the considered scenarios. Cross-border hydrogen infrastructure needs strongly depend on the geographic allocation of renewable energy sources across Europe. Security of supply in Europe can be maintained without investing in additional cross-border methane pipelines. Existing methane pipelines will be repurposed or characterized by low utilisation rates at the 2050 horizon.

**Conclusions: **The multi-energy optimization approach developed is well suited to assess electricity, methane and hydrogen infrastructure projects and their interdependencies considering various scenarios. While electricity and methane infrastructure needs are quite robust across several sensitivities on a climate neutral scenario, hydrogen infrastructure needs are more uncertain and depend on various factors such as the level of hydrogen demand, its competition with biomethane and the level of colocation between RES generation and hydrogen demand.

## Introduction

The European Green Deal sets out the European Commission’s ambitions in tackling climate and environmental-related challenges. The Green Deal targets a 55% reduction in greenhouse gases emissions at the 2030 horizon compared to 1990 levels, and aims at achieving climate neutrality by 2050.

Reaching these targets will require colossal efforts in energy efficiency to reduce the energy demand, in the deployment of decarbonised energy sources, and in infrastructure to enable dynamic interlinkages between sectors and energy vectors.

The Energy System Integration Strategy and Hydrogen Strategy that have been presented by the European Commission during the summer 2020 aim at transforming the currently still siloed design of the European energy sector into a much more integrated system, allowing electrons and molecules to play a complementary role. One of the technologies that interlinks the electricity and gas systems is electrolysis, i.e. the conversion of electricity into hydrogen, estimated to reach up to 500 GW by 2050 in the policy scenarios of the European Commission ^1^ in order to meet the demand for decarbonised gases and fuels.

The European Commission has performed multiple modelling exercises aiming at designing transition pathways, based on different technological options and behavioural assumptions. The Long-Term Strategy, published in November 2018, analyses the role of different technological options. In particular, the so-called 1.5TECH scenario reaches carbon neutrality at the 2050 horizon and foresees a dramatic increase in Renewable Energy Sources (RES) generation capacities compared to current levels, to allow for direct and indirect electrification.

The important deployment of variable renewable energy sources, the interlinkage of methane, hydrogen, electricity and heat, and the flexibility services that can be offered by end-uses will need to be supported by the right type of infrastructure (grids, pipeline, storage), to facilitate full integration and minimize costs. Yet, several infrastructure configurations can be considered (e.g., electrolysis located close to RES generation or close to hydrogen consumption centres, repurposing of existing methane infrastructure to make it compatible with 100% hydrogen, etc.), leading to vastly different investment needs. In all cases, a system-wide, integrated and forward-looking approach is required to identify interdependencies and synergies between vectors and sectors, and provide insights into the optimal level of energy infrastructure to support a 1.5°C-compatible economy.

## Objective of the analysis

This analysis applies a multi-energy modelling framework to evaluate the needs for infrastructure in a 2050 1.5°C-compatible scenario. It builds upon framework assumptions from the European Commission’s 1.5TECH scenario as well as on variations of this scenario based on the Paris Agreement Compatible (PAC) scenarios ^2^ and 1.5LIFE ^3^ scenario of the EU’s Long-Term Strategy (LTS). Many hypotheses are characterised by an important level of uncertainty (e.g., cost of repurposing pipelines, economic case for hydrogen distribution networks, cost of electrolysers, etc.). Therefore, we have focused the analysis on four high-level questions:

1. **Cross-border methane** – Is there a need to reinforce the European methane infrastructure beyond its current level? Due to the change of the structure of gas flows that can be expected at the 2050 horizon, are there pipelines with very low utilisation rates?
2. **Cross-border electricity** – How important is the need for cross-border electricity interconnectors, considering the impacts of electrolysers and their geographical allocation?
3. **Cross-border hydrogen** – Is there a need for cross-border transport of hydrogen? If yes, could part of the existing methane infrastructure be repurposed?
4. **Robustness** – How does the need for infrastructure depend on key assumptions, and how does the (non-)colocation of renewables and hydrogen demand affects infrastructure needs?

In order to provide insights into these questions, we perform a modelling exercise where we jointly optimise the capacity of hydrogen, methane and electricity infrastructure and their use, for a given 1.5°C-compatible scenario at the 2050 horizon, using the Artelys Crystal Super Grid software. We assess the robustness of the conclusions to several key assumptions by performing sensitivity analyses with respect to hydrogen demand levels, bio-methane supply, and wider use of direct electrification to supply low-temperature heat.

This study does not aim at identifying the precise set of infrastructure projects that should be built at the 2050-time horizon, but rather to identify key lessons that can be learned from this exercise, and to translate them into policy recommendations.

This study significantly extends the state-of-the-art of multi-energy simulations. As far as the authors are aware, this exercise is the first one to explore the needs for energy infrastructure with a joint electricity, hydrogen and methane model that maintains an hourly time resolution over the entire year. The use of an hourly time resolution is of primary importance to ensure one captures the impacts of the variability of RES that can heavily impact the operational management of electrolysers and hence the deployment of electrolysers, their location and the infrastructure to transport energy (via electrons or molecules).

## Methods

### A bottom-up optimization approach

The modelling exercise carried out uses the multi-energy system modelling platform Artelys Crystal Super Grid (ACSG). A detailed description of the underlying mathematical system, incl. objective functions, constraints and equations describing asset behaviours used is openly available for download from the METIS website here. METIS was likewise developed by Artelys and relies on the same methodological principles. The approach we apply could be reproduced with an open optimisation model such as PyPSA-Eur-Sec.

In this analysis, a joint model of the European electricity, hydrogen and methane systems was used **.**The model allows for a joint optimisation of investments and operations (with cost minimisation as its objective function) for a given year using an hourly time resolution and a national granularity. All generic data sourced from the METIS website and the data specific to the present analysis is available in *Underlying data* ^1^.

The model is able to simultaneously optimise the operations of and investments in all categories of assets, including different generation technologies, flexible consumption technologies, storage assets, cross-border interconnections and pipelines between areas. The model allows for global constraints to be introduced, such as CO2 budget or maximum output by a given technology over the year (e.g., maximum running hours of coal assets may be constrained).

The costs that are considered in the optimisation include operational costs, i.e. fuel and CO _2_ costs, variable O&M costs and loss of load penalties (if any), as well as investment costs for all technologies subject to capacity optimisation. The model has to ensure that electricity, hydrogen and methane demands are met at any moment in time ^4^ in the considered area (EU27 + Norway, Switzerland, UK, Macedonia, Montenegro, Serbia and Bosnia-Herzegovina).

The catalogue of investment options considered in the present analysis is composed of the following assets:

□ Cross-border infrastructure for electricity, hydrogen (incl. via repurposing) and methane
□ Hydrogen storage assets
□ Electrolysis, steam methane reformers (SMR) and methanation assets
□ Batteries and pumped-hydro storage assets
□ Gas power plants (combined cycle gas turbines, CCGTs, and open-cycle gas turbines, OCGTs)

Several demand-side flexibilities are included in the modelling of the European power system:

□ The charging patterns of electric vehicles are optimised, depending on the user profiles (home charging/work-charging).
□ The operation of residential and tertiary heat pumps is simulated by optimising the heat production from heat pumps (considering the impacts of the temperature on the effective coefficient of performance, COP, of heat-pumps), the operation of thermal storage and the heat production by electric back-up heaters ^5^.

**Figure 1.**
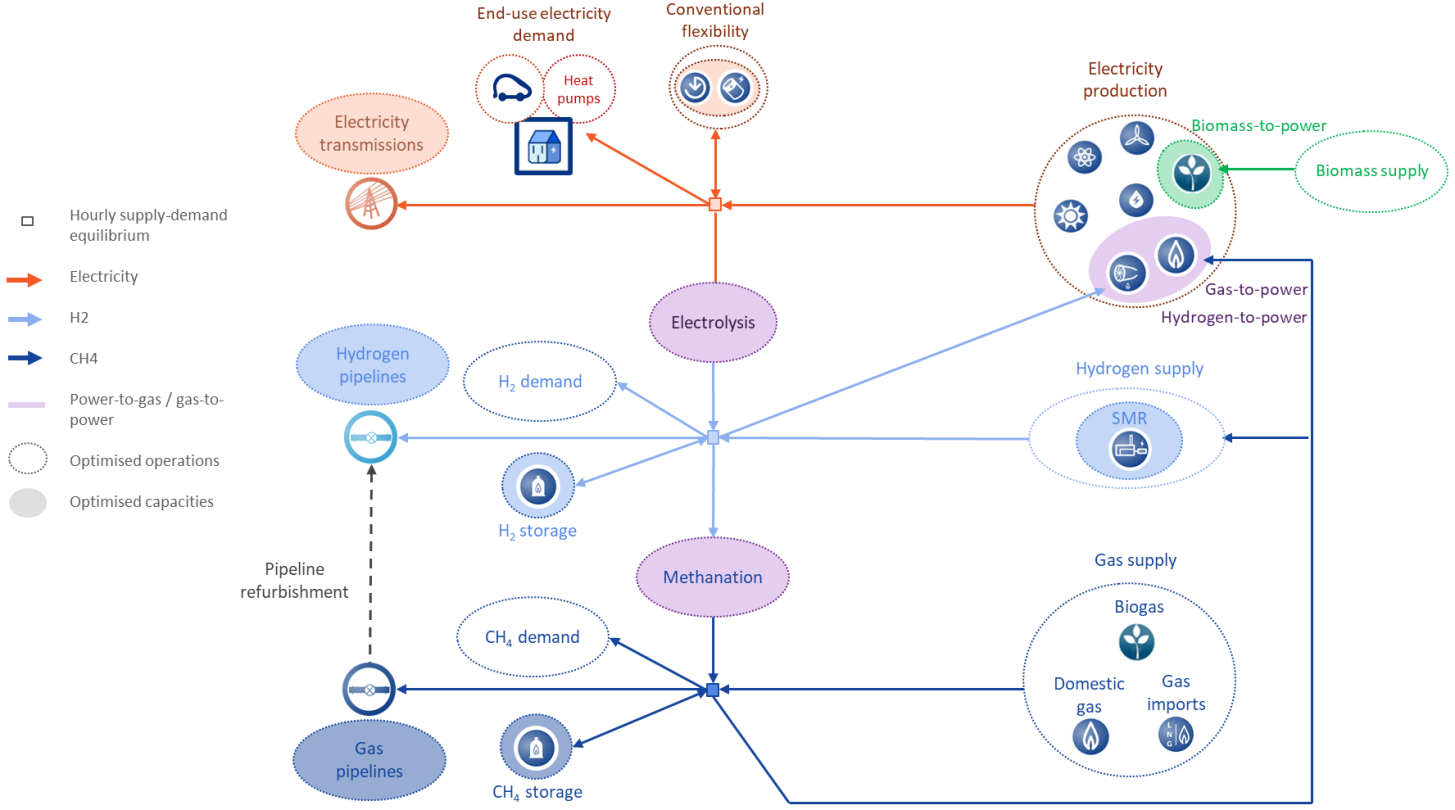
Schematic overview of the modelling structure of the European power, gas and hydrogen systems. H2, hydrogen; CH4, methane; SMR, steam methane reformers.

Figure 1 provides an overview of all the types of assets that are represented in the model and indicates whether they are subject to dispatch optimisation only (highlighted by a dotted circle) or capacity and dispatch optimisation (highlighted by a shaded circle).

The results of the optimisation carried out with Artelys Crystal Super Grid include the investments in the previously mentioned investment options and the hourly dispatch of the different system components of the system (power generation, storage, interconnections, flexible consumers etc.). The Artelys Crystal solution further delivers a set of pre-defined key performance indicators (KPIs), such as total investment and production costs, CO2 emissions, RES curtailment, and security of supply indicators. The full list of KPIs is available in our online documentation.

### Modelling the reference scenario

The reference scenario is largely inspired by the 1.5TECH scenario for 2050 developed by the European Commission in its 2018 Long-Term Strategy ^6^. The main hypotheses adopted from this scenario include (all figures corresponding to EU27+UK geographic scope):

□ **Demand levels**: circa 4000 TWh of final electricity demand (including 500 TWh for electric vehicles and 250 TWh for heat pumps), 1600 TWh of final hydrogen demand and 1200 TWh of final methane demand. Additional losses from the system of 6% is added to the electricity overall consumption.
□ **Electricity supply**: variable renewable power generation (vRES) as main source with circa 1000 GW of solar PV, 750 GW of onshore wind and 450 GW of offshore. The thermal fleets complete the capacity mix with around 120 GW of nuclear capacity, 50 GW of bioenergy capacities with carbon capture and storage (BECCS) and 135 GW of fossil fuel-based capacities (including 90 GW of gas-fired capacities and 45 GW of coal and oil-fired capacities, mainly used as reserve).
□ **Biomethane supply**: 825 TWh

**Figure 2.**
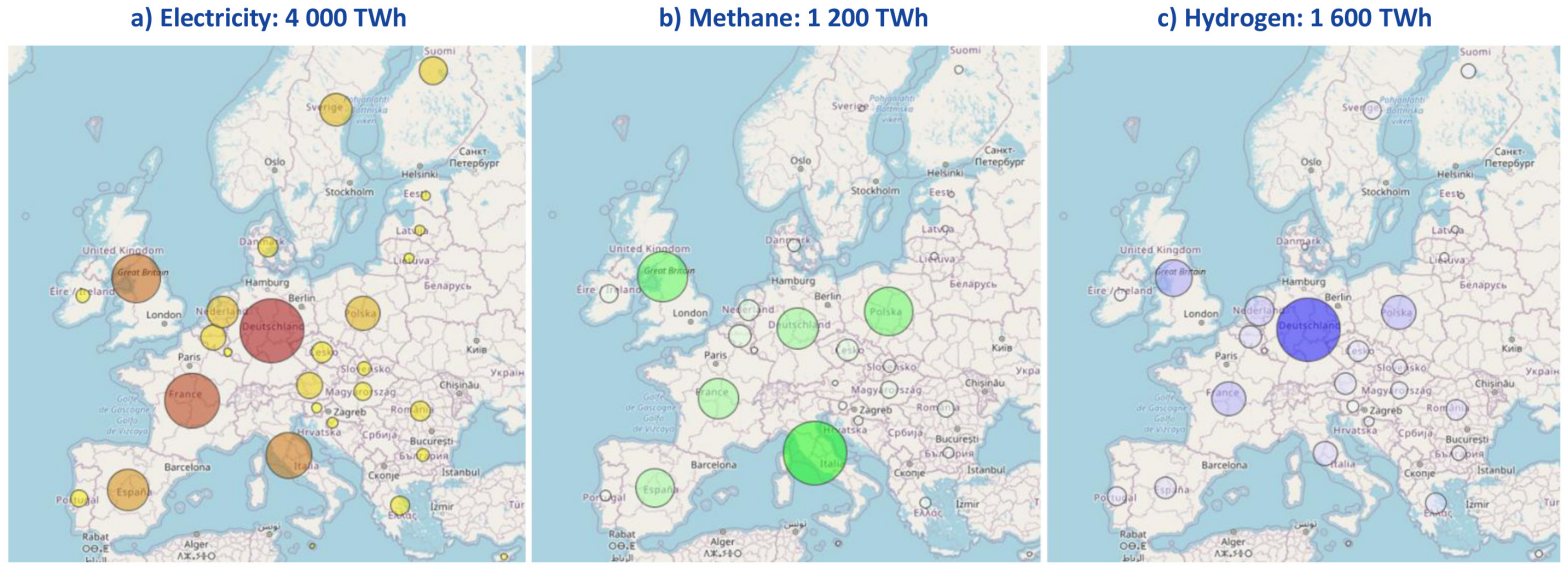
Final energy demand breakdown in the reference scenario.

Since the country-level assumptions of the LTS pathways have not been made publicly available by the European Commission, we have developed a disaggregation methodology to generate a country-level scenario. The disaggregation methodology is based on the following principles:

□ Adopt the EU-wide assumptions of the LTS 1.5TECH scenario (e.g. total demand by fuel, total installed capacity for each technology, etc.).
□ Disaggregate these assumptions at country level using distribution keys (cf. Figure 2 for the breakdown of final energy demand as an example). In practice, most of the distribution keys are based on the use of country-level assumptions published in the Ten-Year Network Development Plan (TYNDP) 2020 of the European network of transmission system operators for electricity and gas (ENTSO-E and ENTSOG, respectively). The plausibility of the disaggregated figures is then analysed via a literature review (e.g. compatibility with RES potentials, order of magnitude of hydrogen demand compared with other scenarios, etc.).

**Electric interconnections** are optimized starting from the net transfer capacities (NTCs) provided in the 2020 Best Estimate scenario of the TYNDP 2018, representing the current European power grid. The installable capacities are limited to 20 GW per border, as costs and impacts on internal networks become very uncertain for high levels of additional interconnection capacity. Investments costs are based on line-by-line transmission projects included in TYNDP 2018: for each cross-border interconnector, an aggregated cost per MW of additional NTC are calculated (for CAPEX and OPEX).

**Cross-border pipelines** are optimized starting from the “Low” scenario of the TYNDP 2018. Data includes the existing infrastructures in 2018 and projects with ‘Final Investment Decision’ status representing the minimum level of infrastructure development considered for the identification of infrastructure gaps. It has been assumed that both directions of gas interconnectors are able to transport the same capacity by 2050 (the additional costs to enable reverse flows are not taken into account). Investment costs in additional pipelines are based on the transmission project list provided by ENTSOG in the 2018 edition of the TYNDP.

The model used for this study optimises the **repurposing of methane pipelines** and investments in new **hydrogen pipelines**. The costs used were extracted from European Hydrogen Backbone and Hydrogen Generation in Europe. In order to consider refurbishment, the number of pipes was estimated on the basis of GIE’s map for each interconnection. We start from a situation without **hydrogen storage**, letting the model decide whether to invest in such a technology considering a cost of 334 € / MWh of stored hydrogen (value of salt cavern storage from Hydrogen Generation in Europe).

The **commodity prices** are based on different sources, including the ENTSOs’ TYNPD, the World Energy Outlook (WEO) of the International Energy Agency (IEA) and the 2016 Reference scenario from the European Commission. Since the **CO2 price** is a key assumption for the modelling, the figure we have adopted originates from the LTS 1.5TECH scenario and equals 350 €/tCO2. 

### Sensitivity analyses

Three sensitivity analyses have been carried out to test the robustness of the evaluation of the infrastructure needs of the European energy systems, by modifying structural assumptions of the reference scenario. They are governed by the willingness to reach higher ambitions on different aspects of the scenario, with updated assumptions based on the Paris Agreement Compatible (PAC) scenarios, and LTS 1.5LIFE scenario.

***A lower hydrogen demand and a smarter allocation of vRES capacities*.** The objective of this sensitivity is to assess the impacts of two important factors: the level of hydrogen demand in the system and the ability to produce it closer to hydrogen demand centres.

**Figure 3.**
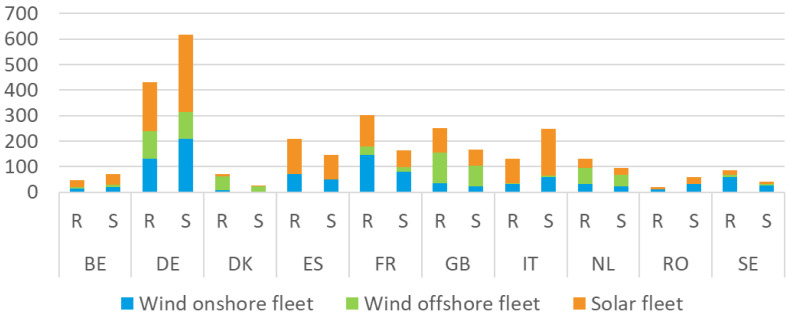
Renewable power generation installed capacities in the reference scenario (R) and the sensitivity (S) for specific countries (Belgium [BE], Germany [DE], Denmark [DK], Spain [ES], France [FR], United Kingdom [GB], Italy [IT], Netherlands [NL], Romania [RO], Sweden [SE]).

This sensitivity analysis assumes a hydrogen consumption that is around 30% lower compared to the one of the reference scenarios, inspired by the PAC scenario’s lower level of hydrogen demand, which is around 1100 TWh across the EU and the UK. The hydrogen demand decrease is shared homogeneously between all countries and associated with a decrease of 600 TWh of renewable power generation (considering an efficiency of 85% for electrolysers). Also, variable RES capacities are reallocated across countries to obtain a better alignment between load centres (for electricity and H2) and power generation volumes, instead of allocating RES to least-cost RES potentials, cf. Figure 3.

***A lower biomethane potential*.** The objective of this sensitivity is to assess the impact of a lower biomethane supply and analyse the impacts of a more local use of biomethane.

**Figure 4.**
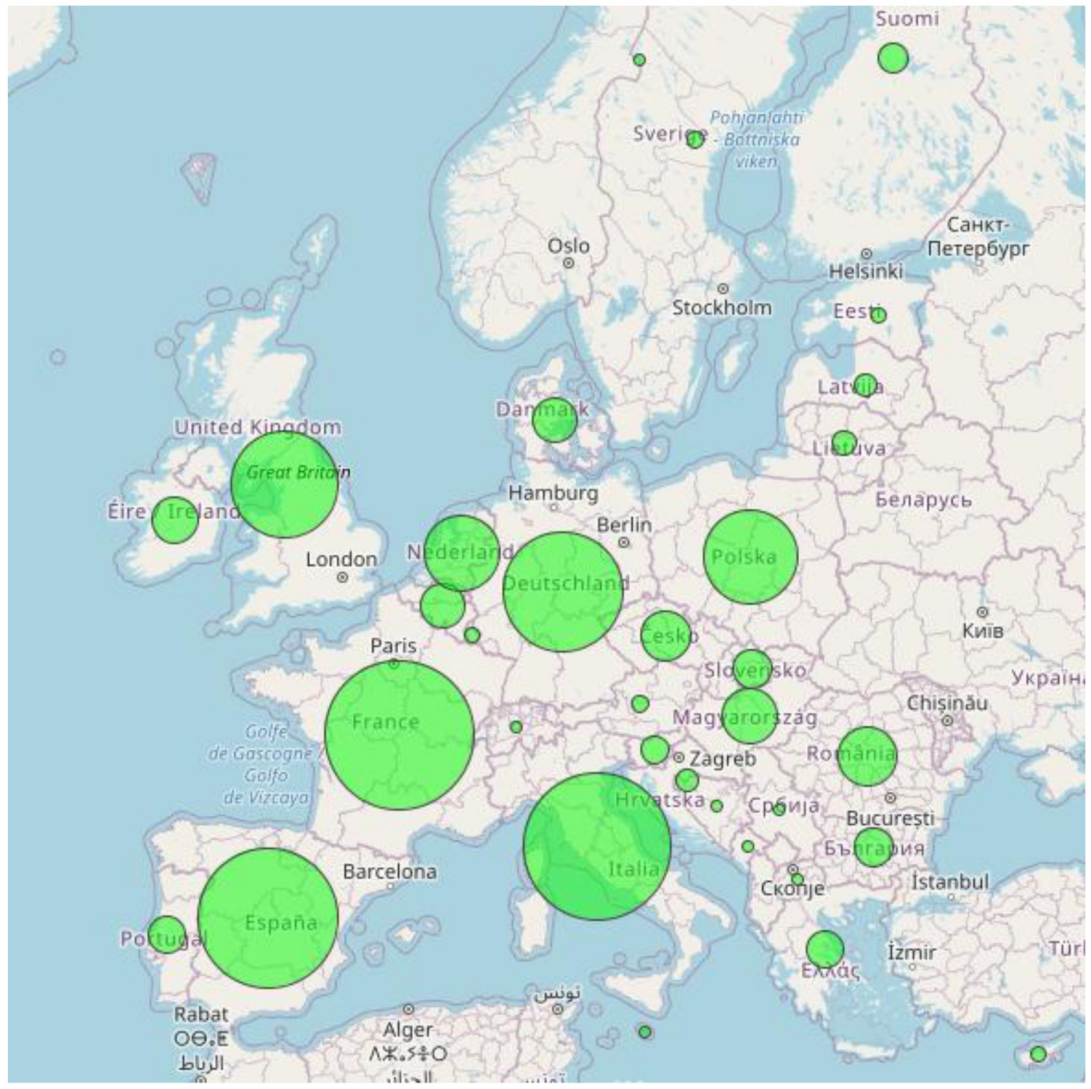
600 TWh biomethane supply breakdown in the sensitivity scenario (EU27+UK).

The second sensitivity analysis assumes a lower biomethane potential at EU level, by reducing the capacity supply to reach the level of the LTS 1.5LIFE scenario: around 600 TWh of biomethane production are assumed in this sensitivity (cf. Figure 4) compared to 825 TWh in the reference scenario.

The biomethane reduction has been performed with the objective of building a more consistent alignment between biomethane supply and methane demand. The reduced biomethane supply potential is compensated by increasing synthetic gas production via electrolysers and methanation plants. Thus, additional renewable capacities are added in the sensitivity in order to cope with the additional demand for carbon neutral power generation induced by the synthetic gas needs. With a respective efficiency of 85% and 79% for electrolyser and methanation plants, around 300 TWh of renewable power generation (wind and solar) are added in this sensitivity.

***A higher energy efficiency and a deeper electrification*.** The objective of this sensitivity is to assess the impact of a deeper electrification of the heat sector on energy infrastructure needs.

It assumes a deeper electrification of the heating end-uses in the residential and tertiary sectors as the remaining gas boilers are replaced by heat pumps, reflecting the PAC scenario assumption of a gas boiler phase out. Since the overall efficiency of producing heat from synthetic-gas-fired boilers is lower than the heat pump efficiency, this sensitivity induces a reduction in electricity demand compared to the reference scenario. In the reference scenario, the gas demand for boilers reaches around 250 TWh. With an 85% efficiency assumption for boilers, the heat demand covered by gas boilers in the reference scenario would reach a little more than 200 TWh. In order to meet this heat demand via the installation of heat pumps, around 50 TWh of additional electricity demand is required. This figure is based on using following assumptions:

□ 95% of this heat demand is covered by heat pumps
□ The remaining heat is provided by an electric back-up heater (during the coldest hours of the year)
□ An average COP of 3.6 is assumed for heat pumps (simulations represent the impact of temperatures on the effective capacity and COP of heat pumps, see e.g. METIS study S6)
□ Electrical heater’s efficiency of 100%

**Figure 5.**
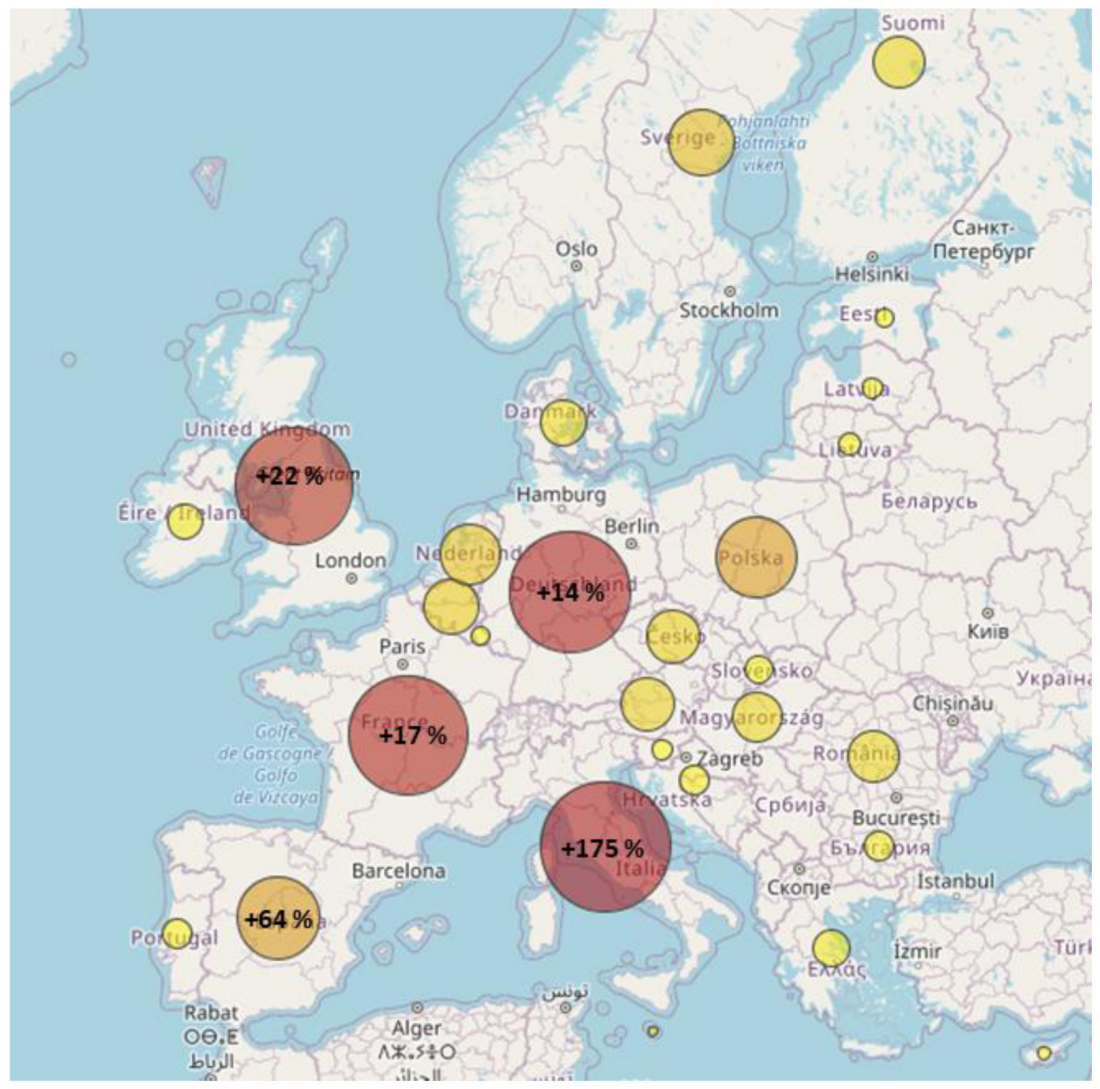
Heat pump demand repartition in the sensitivity and comparison with the reference scenario.

The 200 TWh of heat demand would thus be met with a little more than 50 TWh of electricity consumption from heat pumps. Figure 5 depicts the electricity demand repartition from heat pumps and relative increase compared in the sensitivity compared to the reference scenario.

As with the other sensitivities, the vRES capacities have been updated in order to adapt to the lower electricity consumption (since part of the boilers were using electricity-derived gases). Total RES capacities were reduced by 5% or 100GW compared to the 2 240 GW of the reference scenario.

## Results

In this section, we provide the key findings of the study. These findings have been identified by evaluating the required investments in electricity, methane and hydrogen infrastructure in several 2050 scenarios: a central scenario based on the assumptions of the European Commission’s LTS 1.5TECH scenario and three sensitivity analyses (see previous section for definitions).

### Finding 1 - Major investment levels in electricity infrastructure

The reference scenario as well as all the sensitivities see major investment needs in the electricity infrastructure appear (from 220 GW up to 280 GW additional electricity interconnectors, cf. Table 1 for the result per scenario).

**Table 1.** Additional interconnection capacity (GW) per scenario.

	Additional interconnection capacity (GW)
**Reference scenario**	250
**Hydrogen sensitivity**	220
**Biogas sensitivity**	250
**EE and electrification** **sensitivity**	280

**Figure 6.**
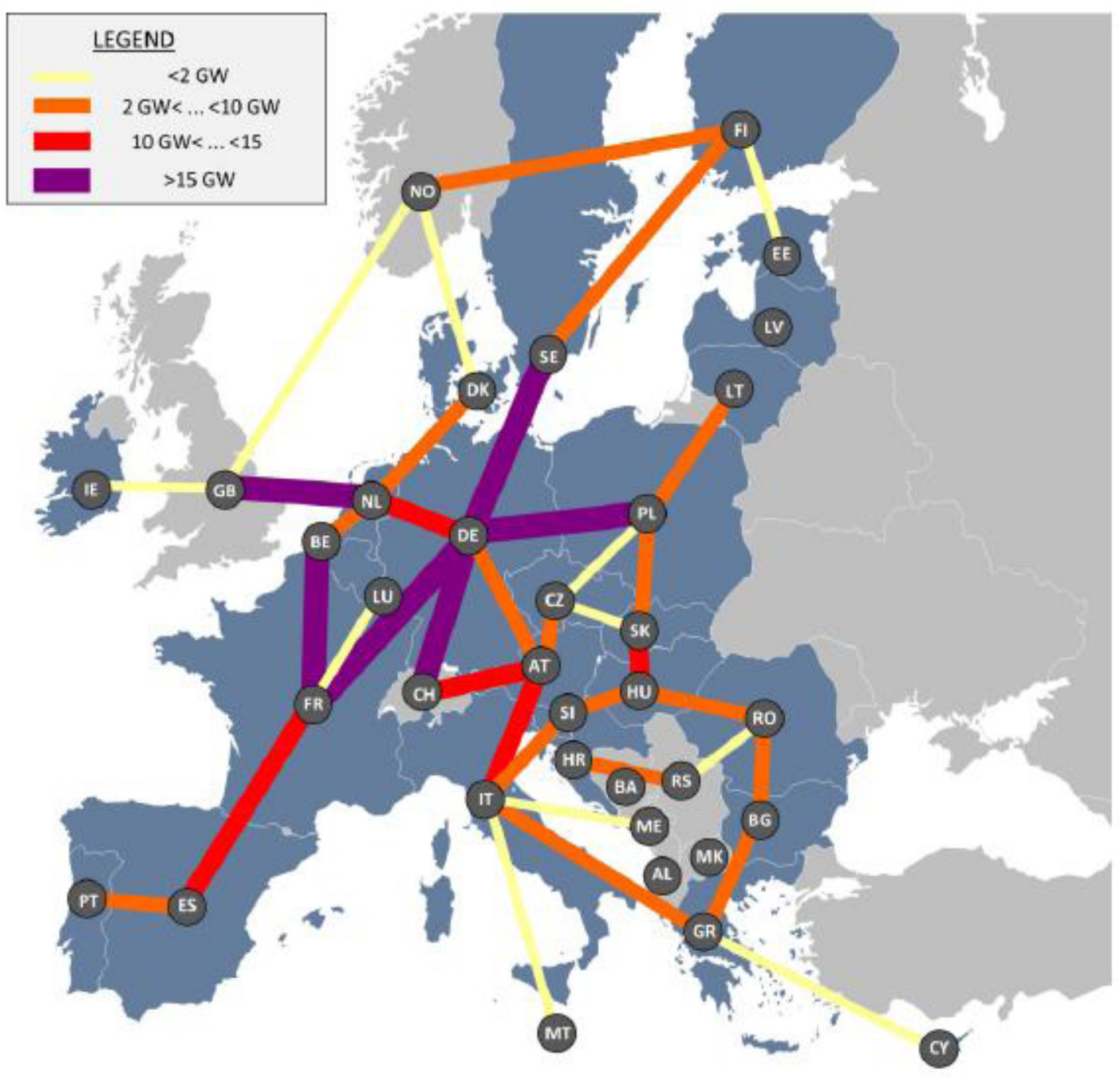
Additional electricity infrastructure in the reference scenario compared to current situation.

Investments would be concentrated around countries that structurally need to import electricity (Italy, Germany, Belgium, Poland, Romania) but also for some countries located on major transit routes (Netherlands, Spain, Switzerland or Austria), as shown in Figure 6.

The key outcome of the hydrogen sensitivity is that the level of required investments in power interconnections can be mitigated by around 10% by a smart distribution of RES capacities in a way that is consistent with hydrogen demand centres.

Given the magnitude of the required investments in electricity infrastructure, a recommendation resulting from this study is that procedures (e.g. related to permitting) should be streamlined and simplified to facilitate investment in cross-border electricity infrastructure.

### Finding 2 - Trade-off between local hydrogen production and infrastructure

**Figure 7.**
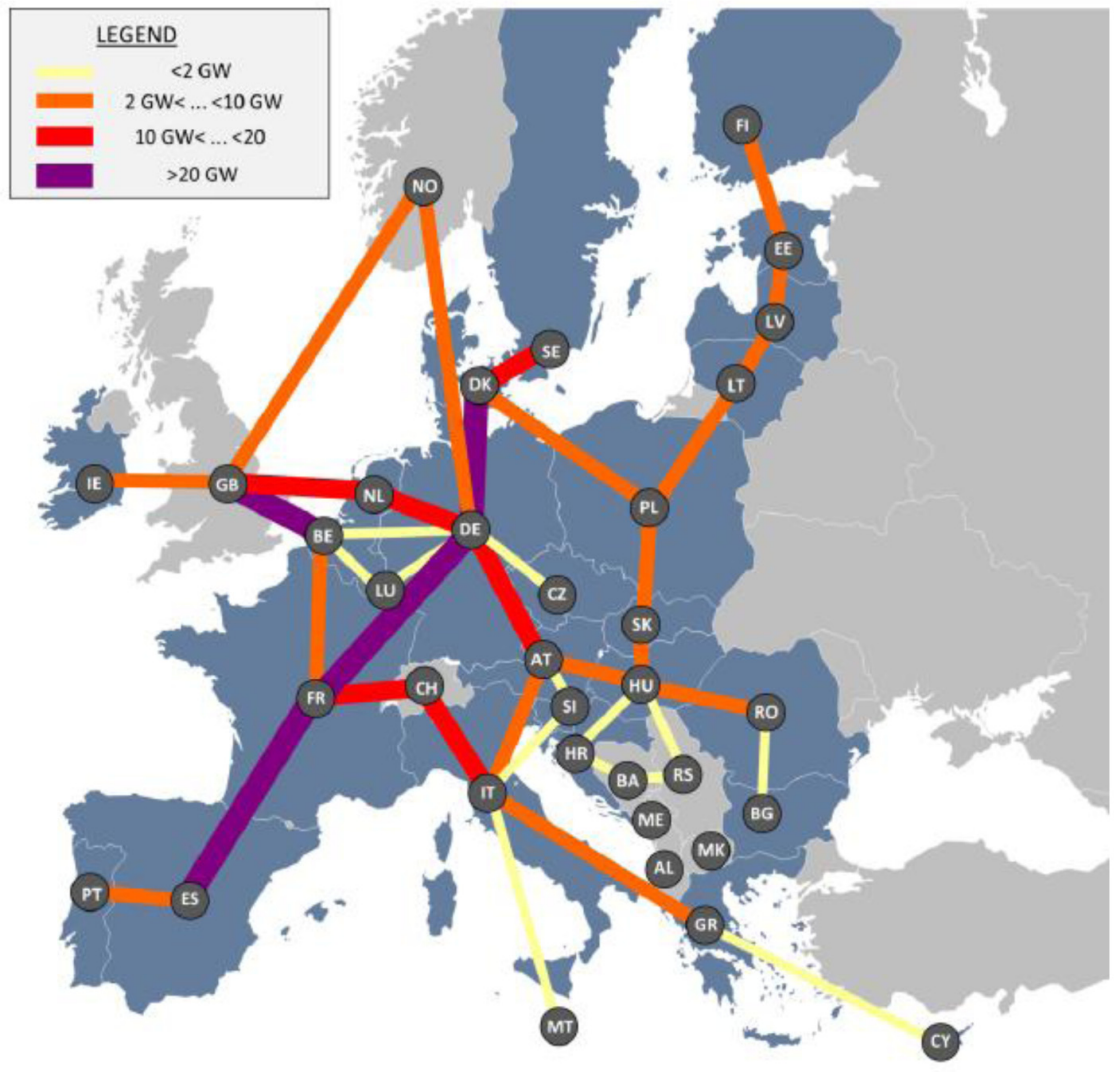
Hydrogen infrastructure in the reference scenario.

Investments in cross-border hydrogen infrastructure will be required in specific areas, notably by repurposing part of the existing methane pipelines in addition to investing in new H2 pipelines. In the reference scenario, we estimate that 320 GW of gas pipeline are repurposed (representing circa 260 GW of hydrogen transmission capacities). Additionally, 310 GW of new hydrogen pipelines are also found to be necessary in the reference scenario. Figure 7 shows the geographical distribution of hydrogen infrastructures.

The hydrogen infrastructure enables to connect main hydrogen exporters (the Netherlands, Spain and France) to the main hydrogen importers (Germany, Italy and Poland).

**Figure 8.**
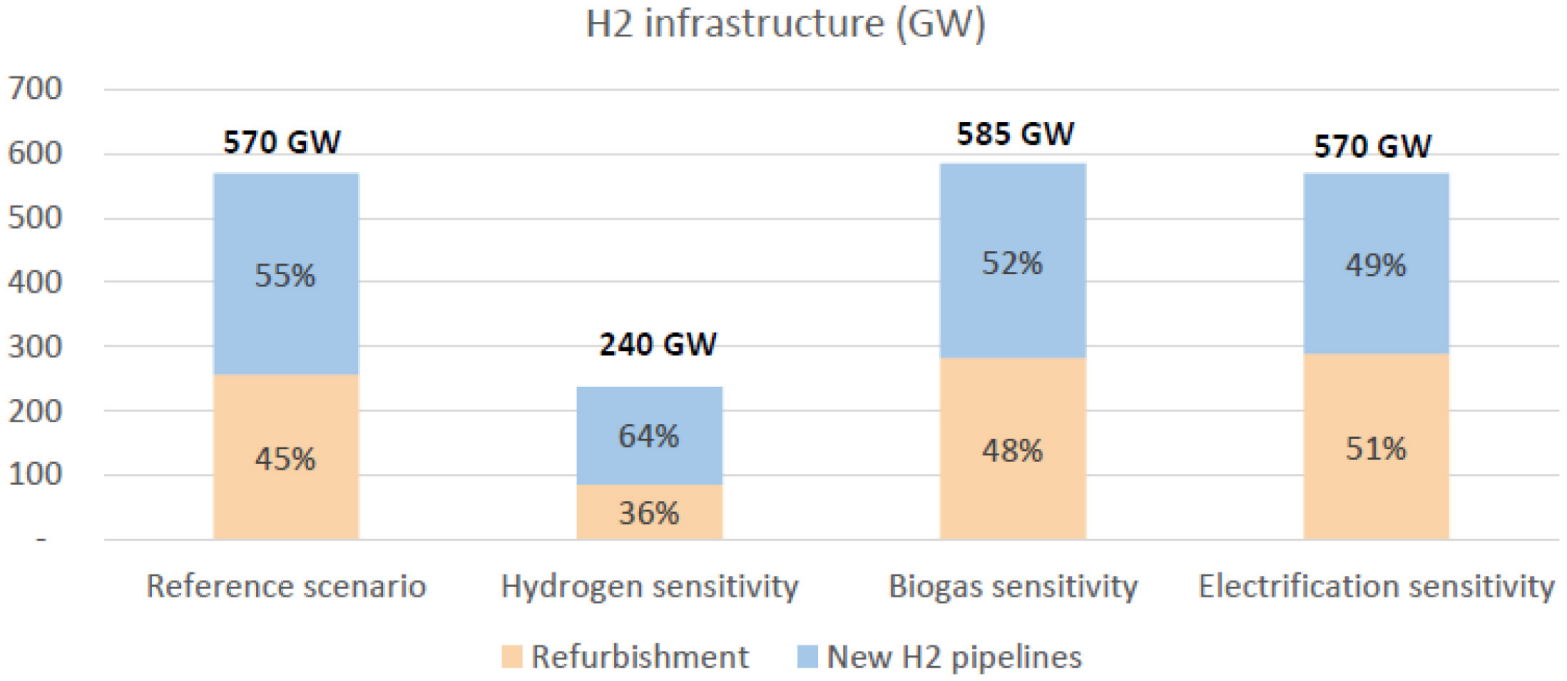
Distribution of investment in H2 (hydrogen) pipelines (distinguished between repurposing and new H2 pipelines for the reference scenario and the 3 sensitivity analyses.

However, the cross-border hydrogen infrastructure requirements are found to be highly dependent on the geographical allocation of renewables in Europe. Our simulations show that the combination of a 30% decrease of hydrogen demand (representing a limitation of the role of hydrogen to hard-to-abate sectors, in line with the PAC scenario) and the reallocation of renewable production sites in proximity to power and H2 demand centres lead to a 60% decrease of the required hydrogen infrastructure (see Figure 8).

Two phenomena are impacting the trade-off between repurposing gas pipelines and building new hydrogen pipes:

□ A lower demand for hydrogen means that one is more likely not to repurpose a given pipeline.
□ A lower demand for methane leaves more room for repurposing as was highlighted in the biogas and electrification sensitivities (see Figure 8).

The key policy recommendation that emerges from this analysis is that scenarios and cost-benefit analyses, CBAs, (especially for power-to-x projects) should examine the impacts of a consistent deployment of RES and electrolysers, in order to avoid unnecessary investments in wires and pipelines.

### Finding 3 - The changing role of methane infrastructure

**Figure 9.**
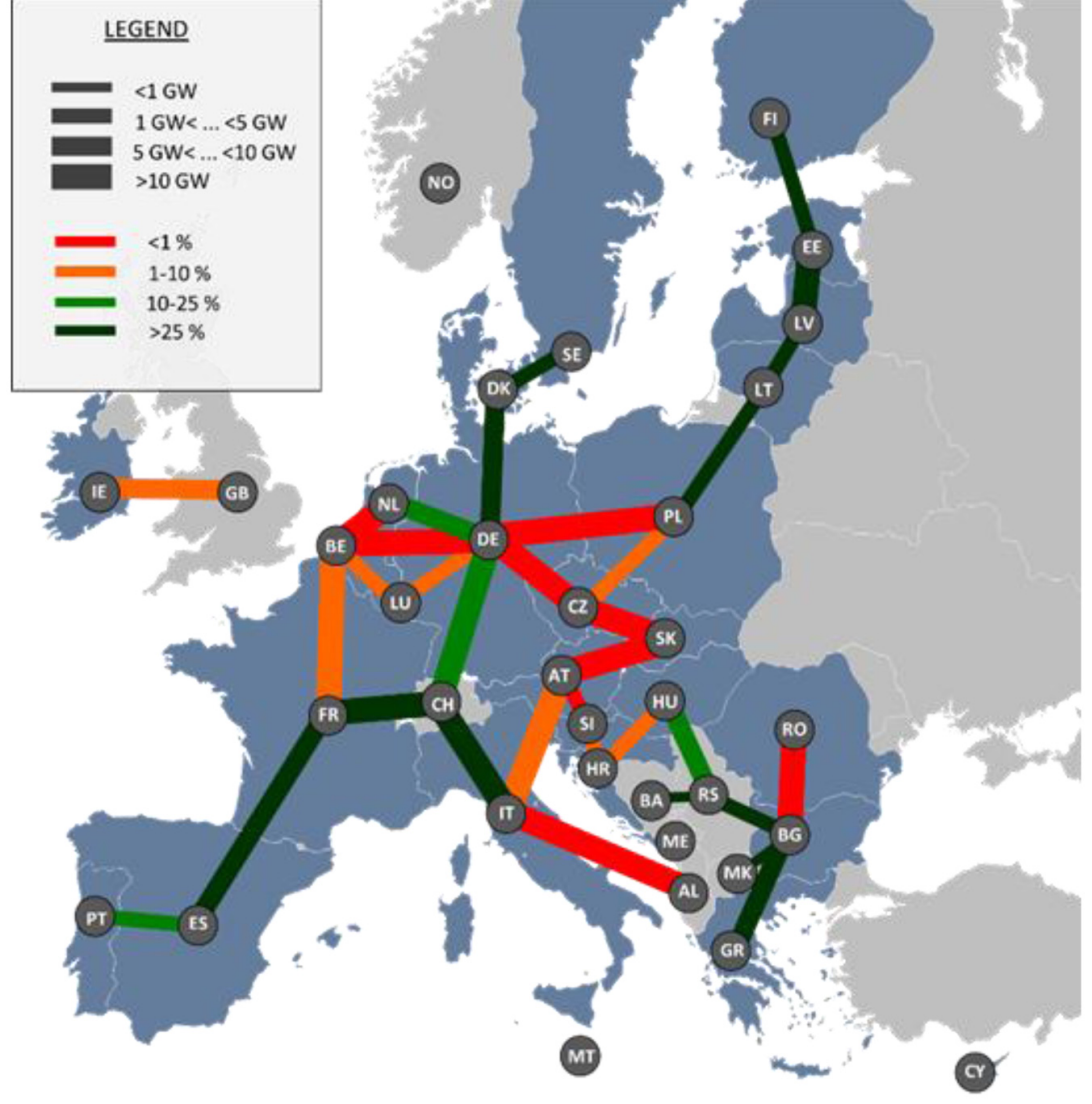
Use rate of gas pipelines in the reference scenario.

Our optimisation results show that there is no need for additional investments in additional methane infrastructure. Indeed, no additional investments are found to be required to ensure security of supply in any of the considered scenarios. As can be seen in Figure 9, part of the existing infrastructure is found to be characterised by low utilisation rates at the 2050 horizon (up to 10 cross-border pipelines with a use rate below 1% over the year) due to the structural evolution of gas flows (a local production combined with the competition between biomethane and hydrogen). Some corridors still remain relevant connecting the north and south to central Europe. The repurposing of part of the existing gas infrastructure is found to be relevant to support the cross-border transport of hydrogen.

The key policy conclusion that comes from this finding is that new methane projects that do not demonstrate benefits in terms of hydrogen transport if repurposed should be excluded. Thus, the evaluation of projects should consider the entire lifetime of the project, considering the possibility to use methane at first stage, and hydrogen in a second phase). Indeed, our results show that new methane infrastructure are unnecessary from a security of supply point of view. Therefore, any additional investments in such infrastructure assets should only be made if they can also serve as hydrogen infrastructure. This transition from transporting methane to transporting hydrogen should be captured when assessing infrastructure projects.

## Conclusions and outlook

We apply a European multi-energy model to assess the required level of cross-border electricity, hydrogen and methane infrastructure in 2050 to ensure the energy demand of a carbon-neutral EU energy system can be met at the lowest cost. The assessment of the reference scenario and the different sensitivities leads to the conclusion cross-border electricity interconnections will be required in any case, whereas there is no need for additional cross-border methane pipelines. The required degree of establishing cross-border hydrogen infrastructure depends on various factors:

□ The level of hydrogen demand, which is highly uncertain. This impacts two aspects: the level of infrastructure and the ratio between new hydrogen and repurposed gas pipelines. Repurposing is a binary process: the lower the hydrogen demand, the less likely it is that repurposing is favoured as the existing gas pipelines have capacities that may be too high compared to the need to transport hydrogen. Investing in a dedicated hydrogen solution might be cheaper in some cases.
□ The level of colocation between RES capacities and hydrogen demand centers. Scenarios and guidelines for cost-benefit analysis should carefully examine the impacts of a consistent deployment of renewable technologies and hydrogen consumption centres in order to avoid unnecessary investments in pipes and wires. Our sensitivities tend to show that hydrogen infrastructure levels can be mitigated by enhancing the degree of colocation between RES production and H2 consumption.
□ The potential competition between biomethane and hydrogen for the use of existing gas pipelines. While there is no need for additional investments in methane infrastructure to ensure security of gas (i.e. methane) supply within the EU, the use of existing gas pipelines will have to be balanced between transportation of methane or hydrogen. If the methane flows remain important, the system needs to keep a sufficient level of methane infrastructure. Reducing the biomethane injection or methane demand and considering biomethane as a local supply - as in the two last sensitivities - will leave more room to repurposing.

From a policy point of view, this analysis demonstrates that the assessment of system needs at the 2050 horizon should be conducted jointly for the electricity, hydrogen and methane systems. For this to be the case, simulation guidelines and tools have to represent all interlinkages within a single framework via an integrated model. Additionally, the repurposing of part of the existing gas infrastructure is found to be a cost-effective way to develop the hydrogen infrastructure and should be considered in the planning of hydrogen infrastructure. Depending on the considered scenarios, the repurposing of gas pipelines could represent between 35% and 50% of the global hydrogen infrastructure. CBA methodology used to assess infrastructure projects should set forward the repurposing of existing pipelines. This advocates for a joint approach to the planning of all infrastructure projects, as decisions related to one vector can heavily impact the value brought by projects transporting another of the energy vectors.

The analysis framework that has been developed could support further analysis of infrastructure needs, and in particular the construction of entirely new transition pathways.

## Data availability

### Underlying data

A detailed description of the underlying mathematical system used, including objective functions, constraints and equations describing asset behaviours is openly available for download from the METIS website here: https://energy.ec.europa.eu/document/download/de87d30a-1915-4ba9-b900-50456ea213b1_en

All generic data (which applies to all scenarios and is independent from the present study context), such as techno-economic data or time series for demand profiles, the availability of power plants or temperature data is contained in the data package available here: https://energy.ec.europa.eu/metis-scripts-and-data_en.

Zenodo: What energy infrastructure to support 1.5°C scenarios? - Scenario data. https://doi.org/10.5281/zenodo.6359001 ^1^.

This project contains the following underlying data:

- EMP-E_Data_Arduin et al_v1.0.xlsx (the assumptions relating to the present analysis, including energy demand volumes and installed capacities).

Data are available under the terms of the Creative Commons Attribution 4.0 International license (CC-BY 4.0).

## Footnotes

^1^ SWD (2020) 176 final Stepping up Europe’s 2030 climate ambition - Investing in a climate-neutral future for the benefit of our people - Figure 48

^2^ CAN Europe/European Environmental Bureau: Building a Paris Agreement Compatible (PAC) energy scenario, June 2020,

^3^ The reader is referred to the LTS reference document for further details about the LTS 1.5LIFE scenario, cf. https://ec.europa.eu/clima/eu-action/climate-strategies-targets/2050-long-term-strategy_en.

^4^ The model accepts loss of load (LoL), yet it comes at high costs, implying that LoL is restricted to 3 hours per year on average.

^5^ Because the coefficient of performance of heat pumps degrades with low outside temperatures, an electric back-up heater is associated to the heat pump, to ensure the heat demand is met cost-efficiently.

^6^ In-depth analysis in support of the Commission Communication COM(2018) 773
