# Supplementary material for: What energy infrastructure will be needed by 2050 in the EU to support 1.5°C scenarios?
Source: F1000Res. 2022 Apr 1;11:387. [Version 1] doi: 10.12688/f1000research.109399.1 (PMC9065927; doi:10.12688/f1000research.109399.1)
Supplement: Peer review history

# Peer review: Reviewer response for version 1

**DOI:** 10.5256/f1000research.120894.r135614

**Published:** 2022-05-03

**Authors:** Ilija Batas-Bjelić

**Affiliations:** 1 Institute of Technical Sciences of Serbian Academy of Sciences and Arts, Belgrade, Serbia

To my knowledge, this is only constrained multi-energy interconnector joint capacity and operation optimization study at EU former/future member states level, and therefore all conclusions should be carried carefully. The electricity interconnection increase (for 250-280 GW) clearly illustrates the electrification of all sectors as the first step of decarbonisation.

 Although the approach is directed toward identifying interdependencies and synergies between energy vectors and sectors, the presented conclusions regarding optimal levels of energy infrastructure are rather particular and not applicable to regions other than Europe. The conclusions are somewhat robust, which at least lead to the identification of key lessons for policy recommendations at EU level.

 There are studies e.g. covering operation optimization at the higher renewable energy penetrations in more details (Pfeifer *et al.*, 2021 ^1^),  but they lack proven effect of interconnection on the relaxation of the total system costs as goal function. Maybe the importance of interconnection contribution should be stressed stronger.

 The loss of load maximum hours and maximum interconnection are bounded smartly to narrow the optimization search space. One of the weak spots of this research might be the utilization of some cross-border pipelines at a rate of less than 1% per year. Thus, this has to be added to constraints. The most interesting direction of future research will be the competition between three interconnector energy vectors.

 The article is rich in illustration and open data.

Is the work clearly and accurately presented and does it cite the current literature?

Yes

If applicable, is the statistical analysis and its interpretation appropriate?

Yes

Are all the source data underlying the results available to ensure full reproducibility?

Yes

Is the study design appropriate and is the work technically sound?

Yes

Are the conclusions drawn adequately supported by the results?

Partly

Are sufficient details of methods and analysis provided to allow replication by others?

Yes

Reviewer Expertise:

Simulation based optimization of national energy systems with policy constraints

I confirm that I have read this submission and believe that I have an appropriate level of expertise to confirm that it is of an acceptable scientific standard.

## References

[1] : Flexibility index and decreasing the costs in energy systems with high share of renewable energy. *Energy Conversion and Management* .2021;240: 10.1016/j.enconman.2021.114258 10.1016/j.enconman.2021.114258

# Peer review: Reviewer response for version 1

**DOI:** 10.5256/f1000research.120894.r129675

**Published:** 2022-04-06

**Authors:** Sonja Wogrin, Thomas Klatzer

**Affiliations:** 1 Technische Universität Graz, Graz, Austria

This article makes an ambitious attempt to depict the European energy system and optimize the required infrastructure to achieve 1.5°C scenarios by 2050. Using the METIS model, every country is represented in an aggregated form (by one node) and sectors gas (hydrogen and methane are considered separately) and power are represented explicitly. Cross-border power lines and pipelines are assessed and conclusions about the EU’s energy infrastructure are presented. The article addresses the important challenge of achieving climate neutrality in Europe by 2050 and is a valuable foundation for future discussions.

 Please find some more detailed comments and questions below:

 It would be interesting to include a brief description of the METIS model formulation. What are the most prominent constraints that have been accounted for by sector? For example, is the power flow considered, or is it a transport problem, what about gas flow equations, etc?

 With respect to the model, the main simplifications should be clearly stated, and it would be interesting to have your opinion on the impact of such simplifications on model outcomes. Are there plans to make an open-source version of METIS available to the public?

 It would improve the paper if you have a summary of the main data, and in what exact granularity you found the data in the source. And most importantly, if data is only available in an aggregated format, how did you specify it. You mention this for the regionalization of energy demand for example, but I am particularly interested in how you obtained hourly granularity for such data. Could you please expand on that? Because METIS runs on an hourly basis but public data sources might now have the same granularity.

 With respect to your findings. In Finding 1, it seems intuitive that a massive expansion of the electric power grid is the cheapest option to achieve climate neutrality. However, there are some bottlenecks here, which should be mentioned. You have discussed permits, but what about political and economic considerations. Often, when cross-country transmission lines are not built, it is not (only) a permit issue. There might be winners/losers of this transmission line, so while I agree that this is technically a great option, there are political complications that should be discussed.

 You mention “variable RES capacities are reallocated .. to obtain a better alignment between load centers”. It seems artificial to re-allocate RES closer to load centers. I think it is fair to say that generated power has the highest value for the system (and also for the investors). I am doubtful that this would happen in reality.

Is the work clearly and accurately presented and does it cite the current literature?

Yes

If applicable, is the statistical analysis and its interpretation appropriate?

Yes

Are all the source data underlying the results available to ensure full reproducibility?

Partly

Is the study design appropriate and is the work technically sound?

Yes

Are the conclusions drawn adequately supported by the results?

Yes

Are sufficient details of methods and analysis provided to allow replication by others?

Yes

Reviewer Expertise:

mathematical modeling and optimization of energy systems; techno-economic analyses of energy systems.

We confirm that we have read this submission and believe that we have an appropriate level of expertise to confirm that it is of an acceptable scientific standard.
